# Neuronal Transcriptome from *C9orf72* Repeat Expanded Human Tissue is Associated with Loss of C9orf72 Function

**Published:** 2020-08-21

**Authors:** Elaine Y. Liu, Jenny Russ, Edward B. Lee

**Affiliations:** 1 Translational Neuropathology Research Laboratory, University of Pennsylvania, Philadelphia, PA, USA

**Keywords:** Dementia, Transcriptome, Frontotemporal, Amyotrophic lateral sclerosis, *C9orf72*, TDP-43, FACS, RNA-seq, Repeat expansion, DENN, Frontotemporal lobar degeneration, Frontotemporal dementia

## Abstract

A hexanucleotide G_4_C_2_ repeat expansion in *C9orf72* is the most common genetic cause of familial and sporadic cases of amyotrophic lateral sclerosis (ALS) and frontotemporal degeneration (FTD). The mutation is associated with a reduction of C9orf72 protein and accumulation of toxic RNA and dipeptide repeat aggregates. The accumulation of toxic RNA has been proposed to sequester RNA binding proteins thereby altering RNA processing, consistent with previous transcriptome studies that have shown that the *C9orf72* repeat expansion is linked to abundant splicing alterations and transcriptome changes. Here, we used a subcellular fractionation method and FACS to enrich for neuronal nuclei from *C9orf72* repeat expanded *post-mortem* human ALS/FTD brains, and to remove neuronal nuclei with TDP-43 pathology which are observed in nearly all symptomatic *C9orf72* repeat expanded cases. We show that the *C9orf72* expansion is associated with relatively mild gene expression changes. Dysregulated genes were enriched for vesicle transport pathways, which is consistent with the known functions of C9orf72 protein. Further analysis suggests that the *C9orf72* transcriptome is not driven by toxic RNA but is rather shaped by the depletion of pathologic TDP-43 nuclei and the loss of *C9orf72* expression. These findings argue against RNA binding protein sequestration in neurons as a major contributor to *C9orf72* mediated toxicity.

## Introduction

Amyotrophic lateral sclerosis (ALS) and frontotemporal degeneration (FTD) are fatal neurodegenerative diseases with overlapping clinical, pathologic and genetic features. ALS is a motor neuron disease that primarily affects the upper and lower motor neurons in the motor cortex and spinal cord, respectively, whereas FTD affects the frontal and temporal lobes, thereby affecting cognition. The most common genetic cause of familial and sporadic cases of ALS and FTD was identified as a hexanucleotide G_4_C_2_ repeat expansion found in the gene *C9orf72* (1, 2). The repeat expansion is found in the first intron of *C9orf72* and can be transcribed into pre-mRNA that contains the repeat. C9orf72 protein has been predicted to be a DENN Rab GTPase, and shown to be involved in vesicular trafficking (3–8). Three potential mechanisms of toxicity have been implicated in contributing to disease: haploinsufficiency due to reduction of C9orf72 protein; RNA foci and titration of RNA binding proteins; and dipeptide repeat protein aggregates (reviewed in (9)). It is currently unclear which mechanism contributes most to toxicity.

Gain of toxic function via RNA foci or dipeptide repeat proteins have been seen in cellular and animal models. Indeed, several RNA binding proteins have been shown to colocalize with repeat containing RNA within RNA foci, suggesting that RNA binding protein titration may be mediating toxicity (10–12). Models overexpressing dipeptide repeat proteins and/or mutant RNA in both cell and animal systems have shown that the repeat expansion and dipeptide proteins may confer toxicity via a variety of pathways including nucleolar stress, nucleocytoplasmic transport defects, heterochromatin abnormalities, and DNA damage (13–32). However, RNA foci and dipeptide repeat proteins do not correlate with neurodegeneration in human tissues and are not toxic in some experimental models (13–16, 33–36), raising questions about the relevance of whether these pathologies contribute to disease.

The loss of C9orf72 protein has been substantiated by the fact that *C9orf72* expansion carriers have reduced *C9orf72* mRNA and protein expression (1, 2, 37, 38). Thus, it is possible that loss of C9orf72 protein may contribute to disease. However, knockout mouse models of *C9orf72* do not show neuronal defects but, rather, exhibit an inflammatory phenotype (39–41), although reduction of C9orf72 protein appears to exacerbate phenotypes in mice overexpressing mutant *C9orf72* RNA (42). We have shown that a subset of *C9orf72* expansion carriers exhibit *C9orf72* promoter methylation, and this is associated with reduced C9orf72 associated pathology, later age at death in FTD patients, slower rates of cerebral atrophy, and improved cognitive ability (43–45). Moreover, the gnomAD human genetics database indicates that missense and loss of function *C9orf72* mutations are tolerated and present in control populations (46). Consequently, the pathogenic role of the loss of C9orf72 protein in ALS/FTD is not entirely clear, and overall it remains unclear which mechanism of toxicity contributes most to disease.

Efforts to better understand these mechanisms include transcriptomic studies to determine whether RNA dysregulation can contribute to neurodegeneration. Indeed, frontal cortical and motor neuron transcriptome analysis from ALS patients showed splicing changes, dysregulation of RNA processing pathways, and alternative polyadenylation in sporadic and *C9orf72* expansion carriers (47–49). More recently, a large transcriptome study of *C9orf72* cases found changes in genes linked to vesicular transport (8). These studies are sometimes complicated by the complex cellular composition of brain tissue which includes neuronal subtypes, oligodendrocytes, microglia, astrocytes, vascular cells and other cell types. Laser capture microdissected tissue studies have been very useful (50) but are labor intensive and often are technically difficult due to relatively low yield and preprocessing steps that can affect RNA quality (51). Single cell RNA sequencing methods have recently addressed the issue of complex cellular mixtures within tissue but suffer from low coverage and data sparsity.

We have previously reported the use of fluorescence activated cell sorting (FACS) to identify transcriptome signatures in neuronal nuclei without TDP-43 from *post-mortem* brain in ALS/FTD patients (52). Here, we have used the same method to determine the role of the *C9orf72* repeat expansion in *post-mortem* neurons from ALS/FTD patients. Compared to the profound transcriptome changes associated with the loss of normal nuclear TDP-43 protein (52), we found that the *C9orf72* mutation was associated with relatively mild gene expression changes and mild splicing changes. Moreover, the molecular signature is largely associated with a loss of C9orf72 function, and not a gain of toxic RNA or dipeptide repeat aggregates.

## Materials and Methods

### Clinical and pathologic assessment

Human autopsy tissue was obtained from the University of Pennsylvania Center for Neurodegenerative Disease Research Brain Bank as described (53). Informed consent from next of kin was obtained for every case.

Isolation was performed exactly as previously described (52). Briefly, mid-frontal neocortex of all cases were dounce homogenized using pestil B (Kimble Chase) in 0.25M sucrose and adjusted to final molarity of 1.6M sucrose in TKM. The homogenate was spun on a 1.8M sucrose cushion on the Beckman Coulter XPN-80 ultracentrifuge at 40,000g for 40 minutes at 4°C (Beckman Coulter Inc, Indianapolis, IN, USA). Isolated nuclei were stained with Alexa Fluor 647 conjugated to 2089 (rabbit polyclonal C-terminal anti-TDP-43 antibody, Center for Neurodegenerative Disease Research, University of Pennsylvania), Alexa Fluor 488 conjugated NeuN (EMD Millipore, Billerica, MA, USA), and DAPI (Invitrogen, Carlsbad, CA, USA). Alexa Fluor 647 was conjugated to 2089 according to the APEX Alexa Fluor 647 Antibody labeling kit protocol (Thermo Fisher Scientific, Waltham, MA, USA). Stained nuclei were sorted for single cells containing TDP-43 and NeuN on the BD FACSAria II (BD Biosciences, San Jose, CA, USA) at 20 psi on 100μm nozzle.

### RNA Isolation and RNA-seq Library Generation

Isolation and library generation was done as previously described (52). Briefly, RNA was extracted using the standard protocol within the AllPrep DNA/RNA Micro kit (Qiagen, Germantown, MD, USA). RNA quality from sorted nuclear RNA was determined based on Bioanalyzer Picochip analysis (Agilent, Santa Clara, CA, USA). Isolated RNA was amplified, made into cDNA, sheared, and libraries were made. The library was quantified using the Qubit dsDNA kit (Invitrogen) and Kapa library quantification kit (KapaBiosystems, Boston, MA). cDNA libraries were pooled, clustered on the cBot and subject to 100 or 125 base pairs paired end reads on the HiSeq 2000 or 2500 (Illumina, San Diego, CA, USA).

### Pre-processing, mapping and filtering of RNA-seq data

RNA-seq analysis was done as previously described (52). Briefly, raw sequencing reads were demultiplexed through the UPenn Functional Genomics Core and analyzed for quality control using FastQC. Reads were mapped to the human genome (GRCh38, GENCODE release 22) using STAR and only uniquely mapping reads were selected for further analysis. Ribosomal and mitochondrial reads were removed and SAM files were converted to BAM files using samtools view and BAM files were sorted by coordinate with samtools sort.

### Creation of non-overlapping gene, exon and intron annotations

Annotations were generated as previously described (52). Briefly, annotations were based on the comprehensive gene annotation file of the GENCODE Release 22 (GRCh38.p2). GTF file was loaded into R (Version 3.2.2; R Core Team (2015): “R: A Language and Environment for Statistical Computing”, R Foundation for Statistical Computing, Vienna, Austria) and converted into a TranscriptDb object. From the TranscriptDb object, all annotated Ensembl genes and their exons were pulled out using exonsBy (by=“gene”) and Ensembl gene IDs were replaced by official gene symbols with biomaRt (Version 2.26.1). Genes and introns were defined as previously described in the above chapter. Regions shared by overlapping genes were removed to count reads that map to one gene or to genic elements (exon or intron) from one gene. Exons and introns annotations were used for subsequent analyses in the R package DEXSeq (1.16.10).

### Differential gene and genic element expression analysis

The genomic annotation used for read counting was done as previously described (52). The mapped, filtered RNA sequencing reads were counted using a custom R script including the R packages Rsamtools (1.22.0), GenomicFeatures (1.22.8) and GenomicAlignments (1.6.3). Briefly, sorted BAM files were loaded into R (3.2.2) and the number of reads mapping to genes, exons or introns, was computed.

Genes were analyzed for differential expression using the R package DESeq2 (1.10.1). Analysis was used to determine differences between nuclei from both *C9orf72* expansion cases and non-diseased controls (option design=~experiment in DESeqDataSetFromMatrix tool where experiment is the subject id). Sex effects were removed with the R package sva (3.18.0) according to the DESeq2 vignette. Manual annotation of gene descriptions was performed using gene descriptions on NCBI.

Differential genic element expression analysis was done using DEXSeq (1.16.10). Briefly, the sorted BAM files were used to count the number of reads mapping to exons and introns using the exon and intron annotation generated above and computed using findOverlaps and countSubjectHits. Reads mapping to exon-intron junctions were excluded. Sex was controlled for by including it in the linear model used in the analysis by adding the term “sex:exon”. The following linear models were used full model = ~sample + exon + sex:exon + experiment:exon and reduced model = ~sample + exon + sex:exon + experiment:exon and reduced model = ~sample + exon + sex:exon, where sample is the sample id and experiment the subject id. In addition, dispersions were estimated using the tool estimateDispersions with option fitType=‘local’. Changes in expression were significant if Bonferroni-Hochberg multiple testing adjusted p-values were less than 0.05.

### Alternative splicing analysis

Splicing analysis was performed as previously described (52). Briefly, all FASTQ files were trimmed to 100bp, aligned to GRCh38 using STAR; ribosomal and mitochondrial mapped reads were removed. SAM files were converted to sorted BAM files and rMATS.3.0.9 was run using default parameters with the following options (-t paired –len 100 –c 0.05 – analysis U). Significant alternative splicing events were used if Bonferroni-Hochberg multiple testing adjusted p-values were less than 0.05.

### RNA binding protein CLIP analysis

CLIP analysis was done as previously described (52) using published TDP-43 iCLIP data from SH-SY5Y cells (54) and hnRNP A, A2B1, F, M, U CLIP data from HEK293 cells (55). Briefly, hg18 Bowtie files were converted to FASTQ files, and aligned to hg38. PIPECLIP was run using the python script. Using R, the “GenomicRanges” package with ‘findOverlaps’ option was used to determine which bins had RNA binding protein sites. Chi-square analysis was done to determine whether there was a significant enrichment of bins with RNA binding protein sites within the significantly differentially used bins linked to the *C9orf72* mutation.

### Principal Component Analysis

Principal component analysis was done using the log transformed read count dataset in R with the ‘stats’ package and ‘prcomp’ function. The coordinates were retrieved and used to plot principal component 2 (PC2) vs principal component 1. To determine the relationship between PC2 vs *C9orf72* expression, the coordinates of PC2 for each sample was plotted against the normalized *C9orf72* expression calculated from DESeq2 from each sample. Pearson’s correlation was performed to determine the correlation between PC2 and *C9orf72* expression. Gene ontology analysis of the top 1% of all genes that contribute to PC2 was done using Webgestaldt (56) with the overrepresentation enrichment analysis using the ‘geneontology’ functional database with a FDR corrected significance level < 0.05.

### Methylation Correlation

Methylation levels for the *C9orf72* promoter in patients were determined as previously described (45). Briefly, genomic DNA from the cerebellum was extracted using the Qiagen DNeasy Blood and Tissue kit and subject to overnight digestion with HhaI and HaeIII (double-digested) or just HaeIII (mock) alone. A small aliquot of DNA was amplified using primers flanking the HhaI cutsite within the *C9orf72* promoter region using 2x FastStart SYBR Green Master (Roche) on the ABI StepOnePlus machine. The difference in cycles to threshold amplification between double and mock digested DNA was calculated as methylation values. Spearman’s correlation was calculated using gene counts for each gene and methylation values for each *C9orf72* mutation case. R package ‘lsr’ with ‘correlate’ function (with options corr.method =“spearman” and p.adjust.method=“fdr”) using the Spearman’s correlation and FDR adjusted p-values was used. Only correlations from genes with HUGO gene symbols were calculated and plotted against gene fold change for each gene that was calculated by DESeq2. This was done using both significantly differentially expressed genes linked to the *C9orf72* mutation (DESeq2 FDR p-value < 0.05) and genome-wide using all expressed genes.

## Results

### Fluorescence Activated Cell Sorting and RNA Sequencing of Sorted Neuronal Nuclei

**Fig 1:**
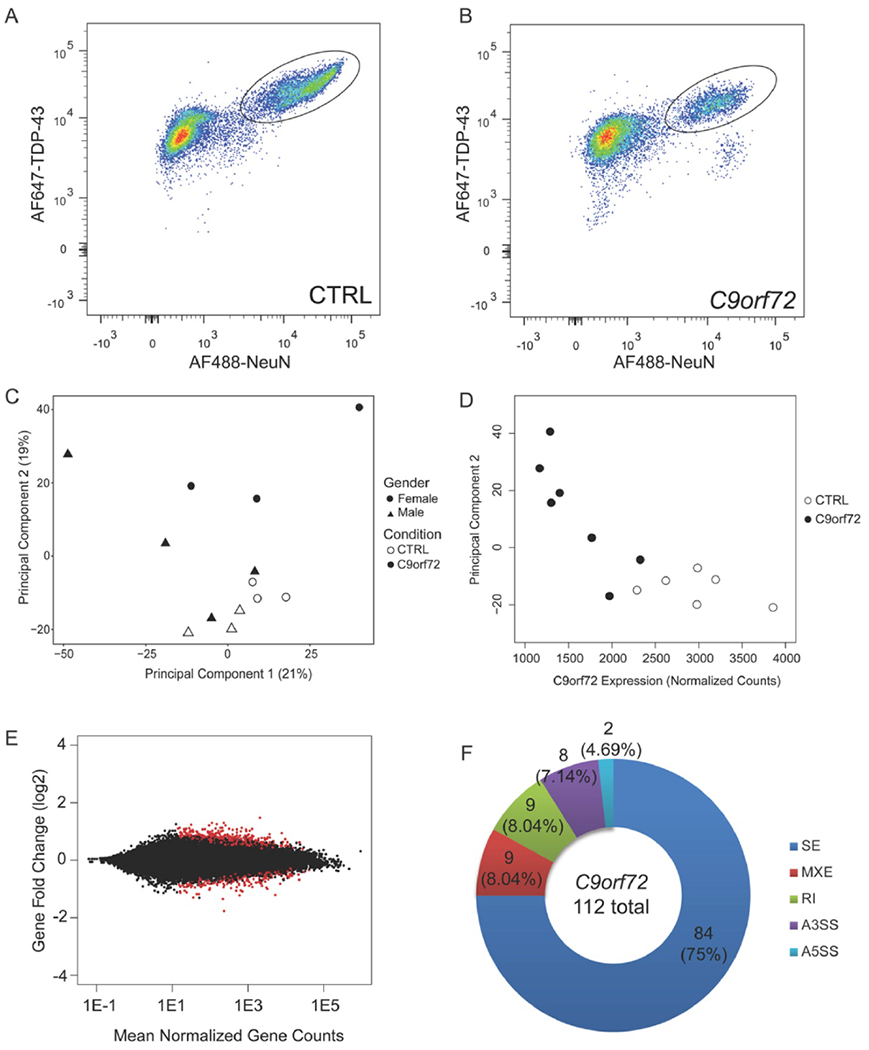
*C9orf72* repeat expansion contributes to relatively mild transcriptome changes. Flow cytometry plots of control (A) and *C9orf72* expansion cases (B) with TDP-43 positive neuronal nuclei circled as collected samples. (C) Principal component analysis using all expressed genes where shape denotes sex and color denotes condition. (D) Principal component 2 strongly correlates with *C9orf72* expression (Pearson’s r = −0.8205; p=0.0006). (E) MA plot showing gene fold change as a function of mean normalized gene counts with red dots being significantly differentially expressed genes. (F) Pie chart distribution of all alternative splicing events between *C9orf72* expansion carriers and non-diseased controls. SE=skipped exons, MXE=mutually exclusive exons, RI=retained intron, A3SS=alternative 3’ splice site, A5SS=alternative 5’ splice site.

The *C9orf72* mutation leads to TDP-43 pathology which includes the formation of neuronal cytoplasmic inclusions and the loss of normal physiologic nuclear TDP-43 protein. We have previously shown that the loss of normal nuclear TDP-43 protein has large effects on the nuclear transcriptome (52). To determine whether the *C9orf72* mutation leads to transcriptomic alterations independent of TDP-43 pathology, we isolated neuronal nuclei with intact TDP-43 expression. Mid-frontal neocortex from 7 *post-mortem C9orf72* expansion carriers and 6 neurologically normal controls were used to isolate nuclei for transcriptome-wide analysis. The autopsy cohort characteristics can be found in Table 1. Nuclei were immunostained for NeuN and TDP-43 and subjected to FAC sorting to isolate NeuN positive, TDP-43 positive nuclei from controls (circled, Fig 1A) and *C9orf72* expansion cases (circled, Fig 1B). RNA from equal numbers of TDP-43 positive neuronal nuclei (35,000-100,000) was extracted and amplified to generate barcoded cDNA libraries for 100 or 125bp paired end sequencing on the Illumina HiSeq 2000/2500. Sequences were mapped to the human genome (Gencode GRCh38) using STAR algorithms (version 2.2.4). There were 1.8 billion reads of which 1.065 billion reads mapped uniquely between the 13 libraries, with an average of 90 million uniquely mapped reads per library. These reads were filtered to remove ribosomal and mitochondrial reads and the resulting reads were used for downstream analysis to evaluate differential expression of genes, genic elements and alternative splicing.

### Functional transcriptomic alterations associated with the *C9orf72* hexanucleotide repeat expansion

To verify that the variation within the dataset is linked to the mutation status, principal component analysis (PCA) was performed using the read counts of the entire dataset (Fig 1C). Principal component #1 (PC1) explained 21% of the variation whereas PC2 explained 19% of the variation (Fig 1C). Based on the clustering of the dataset, sex explains the variation in PC1 while the *C9orf72* repeat expansion explains the variation in PC2. Indeed, PC2 strongly correlated with *C9orf72* mRNA expression (Pearson’s r=−0.8205; p=0.0006), suggesting that *C9orf72* expression is the major underlying biological variable that contributes to the variation in PC2 (Fig 1D). To identify whether there were enriched pathways within the genes that contribute most to PC2, gene ontology analysis was performed using the top 1% of all expressed genes (318 genes) that contribute to PC2. One pathway that is of interest is the cytoplasmic vesicle part or cytoplasmic vesicle membrane (Table 2) and is consistent with the functional role of C9orf72 as a vesicle trafficking protein and Rab GTPase (3, 5–7).

Previous studies have found that the G_4_C_2_ RNA foci may colocalize with different RNA binding proteins (RBPs), suggesting that sequestration of these RBPs can result in aberrant RNA processing (10–12). Differential gene expression analysis showed that there are 323 significantly differentially expressed genes with 202 upregulated and 121 downregulated genes linked to the *C9orf72* mutation (Fig 1E, supplemental table 1). Similar to a previous transcriptome analysis on *C9orf72* expansion carriers on the frontal cortex, there were more upregulated genes than downregulated genes within the differentially expressed genes (49). Notably, the number of differentially expressed genes linked the *C9orf72* repeat expansion was relatively mild compared to the massive transcriptome-wide alterations we previously described associated with TDP-43 pathology in these same cases (5,576 significantly differentially expressed genes, described in (52)).

Further annotation shows that these genes are generally involved in synaptic vesicle fusion or vesicle formation (Table 3). Additional genes related to vesicle transport and endosomal trafficking are also dysregulated. Interestingly, nine out of these 11 genes are upregulated. Additional genes that were upregulated were involved in protein aggregation. *DNAJB2* is almost exclusively expressed in neurons and has been shown to resolve TDP-43 aggregates by interacting with heat shock protein 70 (57). *MGRN1* has been shown to confer cytoprotective effects in an ALS mouse model and can suppress chaperone associated misfolded protein aggregation and toxicity (58). Given that *C9orf72* expansion cases exhibit protein aggregates in the form of dipeptide repeat protein and TDP-43 inclusions, it is possible that these genes are upregulated in response to these aggregates. Lastly, there were also genes that are involved in DNA repair (*FAAP20*, *FAM175A*, *FANCB*, *HMGN1*) (59–62) and response to DNA damage (*KDM4B* and *KIN*) (63–65). Annotation of these genes reveal multiple themes relevant to *C9orf72* including synaptic vesicle formation, endosomal trafficking, chaperone associated protein aggregation, and DNA damage (Table 3).

### *C9orf72* mutation is associated with mild splicing alterations

Previous studies have shown that there are abundant alternative splicing changes that are linked to the *C9orf72* mutation (49). The finding of RNA binding proteins that colocalize with RNA foci also support the idea that RNA binding protein sequestration and subsequent splicing alterations may contribute to mutant *C9orf72* toxicity. To evaluate splicing changes associated with the *C9orf72* mutation, rMATS was used to align junction reads to annotated junctions from GRCh38 assembly. Within our dataset, there were a total of 112 events that were significantly alternatively spliced which included 84 skipped exons (SE), 9 mutually exclusive exons (MXE), 9 retained introns (RI), 8 alternative 3’ splice sites (A3SS), and 2 alternative 5’ splice site (A5SS) events (Fig 1F, supplemental table 2). These events affected 111 genes, of which only 3 were also significantly differentially expressed. Gene ontology analysis of 111 genes did not result in any significant enriched pathways. Therefore, we observed relatively mild splicing changes associated with the *C9orf72* mutation. This may be due in part attributable to technical differences where the use of nuclear RNA in our study likely reduces sensitivity in terms of identifying splicing alterations.

### *C9orf72* mutation is associated with differentially used 3’ UTRs

**Fig 2:**
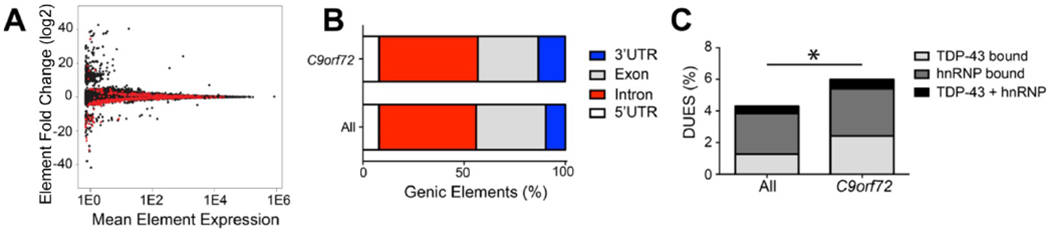
The *C9orf72* mutation is associated with differential usage of genic elements with RNA binding protein sites. (A) MA plot of DEXSeq differentially used genic elements with red being significantly differentially used elements. (B) Genic elements (5’ UTR, exon, intron, 3’ UTR) distribution associated with *C9orf72* mutation and all expressed bins, (c) Differentially used elements that are bound by TDP-43 and hnRNP (χ^2^=5.617; p=0.0178).

Altered RNA binding protein function has been proposed to contribute to *C9orf72* toxicity. RNA binding proteins can bind to different genic elements including exons, introns and untranslated regions (UTR). If RBP activity is altered via titration of these proteins, it is possible the regions that may be bound by RBPs are also differentially expressed. Thus, DEXSeq was used to determine whether the presence of the repeat expansion resulted in differential usage of genic elements. There were a total of 865 significantly differentially used elements linked to the repeat expansion affecting 791 genes. Specifically, there were 610 significantly downregulated elements and 255 significantly upregulated elements (Fig 2A). When the elements were annotated as either 5’ UTR, exon, intron or 3’ UTR, there was a significant enrichment of differentially used 3’ UTRs due to the *C9orf72* mutation (13.35% vs 9.78%, χ^2^=12.50, p=0.0004) (Fig 2B). Given the role of RBPs in binding to 3’ UTRs, we hypothesized that the significantly differentially used elements were enriched for RBP binding sites. Thus, hnRNP binding sites, including that of TDP-43 and other hnRNPs were identified using PIPE-CUP algorithms applied to published data sets (54, 55, 66). Indeed, there was an enrichment of differentially used genic elements that contain TDP-43 and hnRNP binding sites (χ^2^=5.617, p=0.0178, Fig 2C). Thus, the *C9orf72* expansion is associated with some differentially used genic elements with RBP binding sites.

### Transcriptomic signature due to depletion of TDP-43 pathologic nuclei

**Fig 3:**
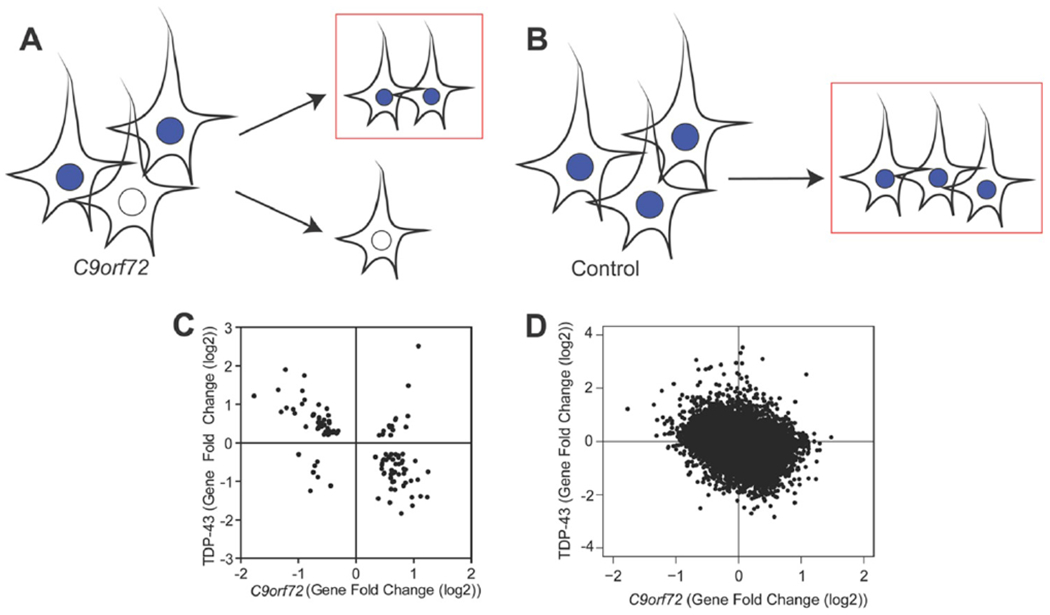
*C9orf72* transcriptome is linked to depletion of TDP-43 pathologic nuclei. (A) In *C9orf72* expansion cases, there are nuclei with TDP-43 (blue) and those without TDP-43 (white). For our transcriptome analysis, only nuclei with TDP-43 were collected (red box), thereby depleting nuclei without TDP-43. (B) In non-diseased controls, nuclei with TDP-43 were collected (red box). (C) Gene fold changes of genes that were common significant differentially expressed linked to TDP-43 loss and the *C9orf72* mutation were plotted, showing that genes that were downregulated due to the *C9orf72* mutation were upregulated due to TDP-43 loss, suggesting that depletion of diseased nuclei (without TDP-43) can partially explain the transcriptomic changes observed in this dataset (Pearson’s r=−0.5708, p<0.0001). (D) Genome-wide gene fold changes that were expressed in both datasets linked to TDP-43 loss and *C9orf72* mutation showed a similar pattern.

Given that neurons with TDP-43 inclusions have died during the course of disease and our experimental design includes removal of neurons with TDP-43 pathology via FAC sorting before RNA sequencing, we next considered the possibility that the transcriptomic alterations associated with the *C9orf72* mutation in this dataset would in part reflect the depletion of neurons vulnerable to TDP-43 proteinopathy. Indeed, within the neocortex, TDP-43 proteinopathy often preferentially affect neurons in superficial cortical laminae (52, 67). As shown in Fig 3A, neurons with TDP-43 pathology including loss of nuclear TDP-43 (white) are depleted in *C9orf72* expansion cases, while control brains have the normal complement of neurons (blue, Fig 3B). As a result, we hypothesized that the depletion of diseased nuclei would contribute to the transcriptomic alterations associated with the *C9orf72* mutation. Thus, we expected that common genes that were downregulated in *C9orf72* expansion cases would be upregulated in nuclei without TDP-43 and vice versa. Indeed, among the 118 common significant differentially expressed genes linked to TDP-43 loss (52) and the *C9orf72* mutation, a majority of them were followed this pattern (Fig 3C, supplemental table 3). A linear regression analysis showed there was a negative correlation between the gene fold change linked to TDP-43 loss and the gene fold change linked to the *C9orf72* mutation (Pearson’s r=−0.5708; p<0.0001). Furthermore, this relationship was extended transcriptome-wide where we observed a negative correlation in all commonly expressed genes derived from both data sets (Fig 3D). For example, *IGSF11* and *PVRL3* expression, preferentially observed in superficial neocortical layers and therefore increased in neuronal nuclei without TDP-43 protein (52), is significantly reduced in the *C9orf72* transcriptome. Conversely, *SYT6* and *COL6A1* expression, preferentially observed in deep neocortical layers and therefore decreased in neuronal nuclei without TDP-43 protein (52), is significantly increased in the *C9orf72* transcriptome. This suggested that depletion of the diseased nuclei in *C9orf72* expansion cases may explain some of the transcriptomic alterations observed in *C9orf72* expansion cases.

### Global *C9orf72* associated transcriptome changes are linked to loss of function of the *C9orf72* protein

Current hypotheses of *C9orf72* toxicity include loss of C9orf72 protein or gain of toxic RNA foci and dipeptide repeat aggregates (9). While some transcriptome changes which appear to be related to *C9orf72* gain of toxic function (genes associated with proteostasis and DNA damage, dysregulation of transcripts with hnRNP 3’UTR binding sites), other changes appeared to be linked to the loss of C9orf72 protein (genes associated with vesicle membranes and endosomal trafficking). However, at a global transcriptome level, it was unclear whether the overall changes associated with the mutation reflect changes linked to a toxic gain of *C9orf72* function or the loss of C9orf72 protein.

**Figure 4:**
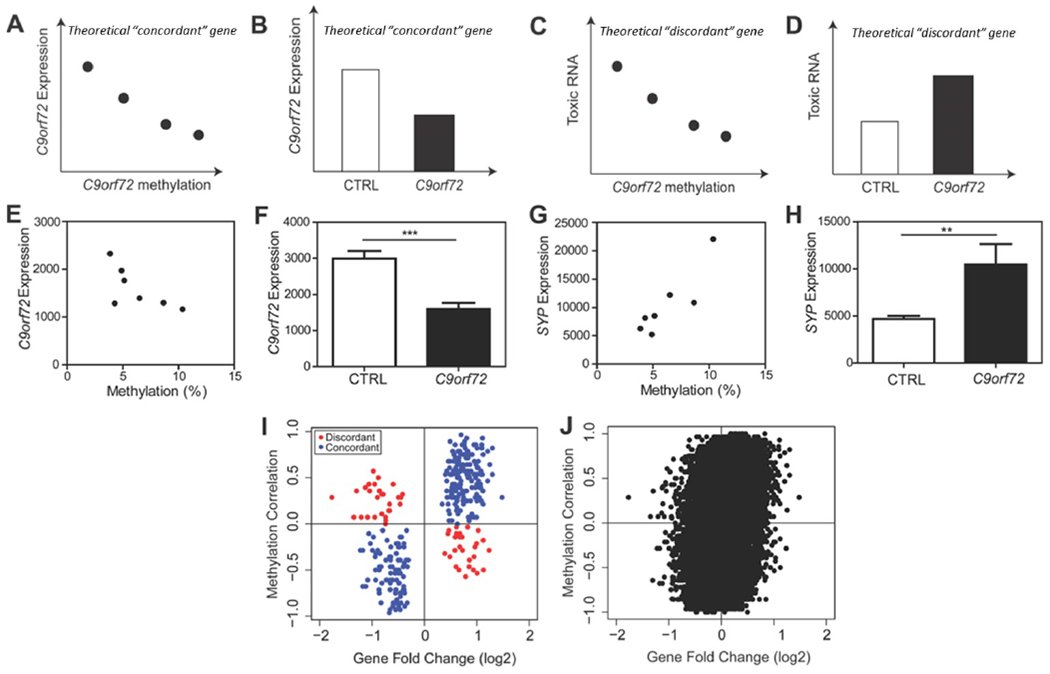
*C9orf72* transcriptome reflects the loss of C9orf72 function. (A) Theoretical analysis within *C9orf72* mutation carriers, we predict that *C9orf72* promoter methylation would negatively correlate with *C9orf72* expression. (B) Theoretical analysis between *C9orf72* mutation carriers and non-diseased controls, we predict that *C9orf72* expression is downregulated compared to controls. This would be an example of a “concordant” gene that suggests this gene is linked to loss of C9orf72 protein. (C) Theoretical analysis within *C9orf72* mutation carriers, we predict that *C9orf72* promoter methylation would negative correlate with toxic RNA. (D) Theoretical analysis between *C9orf72* expansion carriers and non-diseased controls, we predict that toxic RNA expression would be upregulated. This would be an example of a “discordant” gene that suggests this gene is linked to gain of toxic RNA. (E) Within the actual data, there was a negative relationship between *C9orf72* expression and methylation (Pearson r=−0.6958, p=0.0825). (F) *C9orf72* expression is downregulated in C9orf72 expansion carriers compared to controls (t-test, p=0.0003) as predicted in genes associated with the loss of C9orf72 protein. (G) Within *C9orf72* mutation carriers, there was a strong correlation between the expression of *SYP* and *C9orf72* methylation (Pearson r=0.8835, p=0.0083). (H) Between *C9orf72* expansion carriers and controls, there was a significant upregulation of *SYP* in expansion carriers (t-test, p=0.03). (I) Using significantly differentially expressed genes, the methylation correlation *within C9orf72* mutation cases was plotted against gene fold change *between* normal *C9orf72* mutation cases and controls. There were more concordant genes than discordant genes, reflecting that the transcriptome was associated with loss of C9orf72 protein. (H) Genome wide analysis of methylation correlation vs gene fold change shows the same trend.

*C9orf72* RNA foci and dipeptide repeat protein aggregates are seen in *C9orf72* repeat expansion carriers but absent in neurologically normal controls. We have also demonstrated that within *C9orf72* mutation carriers, *C9orf72* promoter methylation is negatively correlated with the accumulation of RNA foci and dipeptide repeat protein aggregates in human brains (43–45). Based on this, we developed a theoretical framework (Fig 4A–D) wherein statistical analysis was performed *within C9orf72* expansion cases versus statistical analysis *between C9orf72* expansion cases and controls. As a theoretical example, using *C9orf72* itself as a gene that contributes to the loss of C9orf72 protein, we predicted that within *C9orf72* expansion carriers, methylation of the *C9orf72* promoter would negatively correlate with *C9orf72* expression (Fig 4A). Moreover, we predicted that between-group analysis would show that *C9orf72* expression is downregulated compared to controls (Fig 4B). This would be an example of a “concordant” gene which provides evidence that the altered gene expression is linked to the loss of C9orf72 protein. Conversely, using similar logic, if a gene contributes to the gain of toxic function such as the formation of toxic RNA, we would predict that within *C9orf72* expansion carriers, methylation of the *C9orf72* promoter would negatively correlate with toxic RNA (Fig 4C). However, between groups, this gene would be upregulated in *C9orf72* expansion carriers compared to controls (Fig 4D). This would be an example of a “discordant” gene, which provides evidence that altered expression of this gene is associated with a gain of toxic function.

Using the actual data, a correlation analysis was performed comparing *C9orf72* expression and methylation of the *C9orf72* promoter within our *C9orf72* expansion cohort (Pearson’s r=−0.6958; p=0.0825) (Fig 4E). Moreover, between-group analysis revealed that *C9orf72* expression is downregulated in *C9orf72* expansion carriers compared to controls as expected (t-test, p=0.0003) (Fig 4F). Another example of a “concordant” gene is synaptophysin (SYP), a synaptic vesicle protein. The same correlation analysis was done which showed a positive correlation within *C9orf72* expansion carriers (Pearson’s r=0.8835; p=0.0083), and an increase in expression in *C9orf72* expansion carriers compared to controls (t-test, p=0.03) (Fig 4G–H, supplemental table 4). Thus, we are able to show that “concordant” genes tend to reflect genes that may be related to C9orf72 protein function.

This analysis was extended to all significantly differentially expressed genes where the “within *C9orf72* mutation cases” methylation correlation value was plotted as a function of “between *C9orf72* cases and controls” gene fold change values (Fig 4I). Across all significantly differentially expressed genes, there were 267 genes found within the concordant quadrants (blue circles) compared to only 56 genes found in the discordant quadrants (red circles) (Fig 4G). Similar results were found when this analysis was further expanded transcriptome-wide, suggesting that the global transcriptome changes linked to the *C9orf72* mutation are associated with loss of C9orf72 protein (Fig 4H). Many “concordant” genes were genes involved in synaptic vesicle formation (i.e. clathrin light chain B, syntaphilin, synaptophysin) and vesicle trafficking (i.e. multivesicular body subunit 12A, RAB40B, member RAS oncogene family). Genes labeled as “discordant” include genes related to inflammatory or apoptotic processes including tec protein tyrosine kinase, tyrosine kinase, and Ring finger protein 152.

Thus two different factors appear to shape this global *C9orf72* transcriptome dataset: (1) the depletion of neurons with TDP-43 pathology prior to RNA-sequencing and (2) the loss of *C9orf72* function. To estimate the relative contributions of these two factors, a multivariate linear regression analysis was performed wherein significant changes in gene expression associated with the *C9orf72* mutation were related to (1) the changes in gene expression we previously identified due to TDP-43 pathology and (2) the loss of *C9orf72* gene expression due to methylation of the *C9orf72* promoter. Together, these two factors explained the majority of the variance in this dataset (R^2^=0.5138; β_methyiation_=0.838, p<2e-16; β_TDP-43_=−0.499, p<2e-16).

## Discussion

Here we report that in using *post-mortem* neuronal nuclei without TDP-43 pathology from *C9orf72* expansion carriers, we were able to identify transcriptome changes that link the *C9orf72* mutation with loss of C9orf72 function. We found that the top 1% of genes that contribute most to changes in *C9orf72* expression were involved in cytoplasmic vesicle trafficking. Significant gene expression changes were linked to the *C9orf72* mutation and featured upregulated genes related to synaptic transmission and endosomal/lysosomal trafficking. Indeed, C9orf72 has been shown to be involved in endosomal or lysosomal trafficking and may be a Rab GTPase, and recent whole tissue transcriptome studies have linked the *C9orf72* mutation to changes in vesicular transport genes (3–8). Nine of the 11 genes related to trafficking were upregulated, potentially reflecting a compensatory mechanism to counteract the loss of C9orf72 and loss of this trafficking related protein.

Further analysis correlating methylation and gene expression changes confirmed that the transcriptome changes are linked to loss of *C9orf72* expression. To parse the relative contributions of C9orf72 protein loss or gain of toxic RNA, a methylation correlation between methylation values and expression data was calculated. This correlation analysis indicated that the transcriptome observed in neuronal nuclei from *C9orf72* expansion cases was driven in large part by the loss of C9orf72 protein as opposed to a toxic gain of function. In contrast, we observed minor splicing alterations and relatively subtle changes in terms of alterations in RNA binding protein function. Thus, the global alterations appear to be most reflective of a loss of C9orf72 function rather than toxic RNA mediated effects. However, it should be noted that C9orf72 methylation was used as a proxy for DPR and RNA foci levels. A more direct analysis of the effects of DPR or RNA foci would require specific isolation of DPR or RNA-foci containing neuronal nuclei which has not yet been possible using FAC sorting approaches. Additional future studies can also include comparisons between ALS/FTD cases with and without the *C9orf72* mutation.

Genes related to DNA repair either as a response to DNA damage or important to employ DNA repair were also dysregulated. Interestingly, recent papers have shown that DNA damage is activated by the *C9orf72* repeat expansion in both ALS patients and experimental models (23, 25, 26, 30, 68). DNA damage has been a consistent feature among trinucleotide repeat expansion diseases and the dysregulation of DNA repair related genes is consistent with DNA damage contributing to neurodegeneration (69). Furthermore, alterations in proteostasis either by failure to fold proteins properly or failure to degrade proteins likely caontribute to protein aggregation and are a common theme among all neurodegenerative diseases (70, 71). Indeed, overexpressing proteostasis factors in animal and cell ALS models prevents protein accumulation and aggregation (57, 72, 73), suggesting that altered proteostasis activity contributes to neurodegeneration. Thus, although the global transcriptome appears to be driven by the loss of *C9orf72* function, embedded within the *C9orf72* transcriptome are changes in gene expression, which may relate to the toxic functions of *C9orf72*.

In comparing the data from previous transcriptome analyses from *C9orf72* expansion carriers, several differences can be observed. Previous transcriptome analyses have identified pathways involved in inflammation and defense responses among dysregulated genes in the frontal cortices from *C9orf72* expansion carriers (49). Within our dataset, we did not find enriched inflammatory pathways, which is likely due to the fact that only neurons were being sequenced. It is possible that because *C9orf72* is expressed in microglia, C9orf72 protein function may have a larger effect on microglial populations (41). Given the evidence supporting that *C9orf72* is involved inflammatory processes, further cell-specific analyses may prove beneficial to better understanding the effects of mutant *C9orf72* on non-neuronal cells in driving disease pathogenesis.

Previous studies have found large numbers of splicing changes in *C9orf72* expansion carriers and found motifs within these splicing changes that correspond to hnRNP H binding (49). However, we did not detect such large changes and instead found milder splicing changes with motifs that do not correspond to any known RNA foci colocalization partners (data not shown). Current studies debated over the disease relevance of RNA foci as some animal models overexpressing the repeat induce RNA foci but do not show motor or cognitive defects (13–16). The lack of splicing changes in our dataset does not support the model wherein neuronal toxicity is linked to the titration of RNA binding proteins within RNA foci.

Overall, our findings argue against RNA toxicity attributed to sequestration of RNA binding proteins. Other disease-related pathways, such as protein aggregation and DNA repair, were observed in this dataset, supporting a potential role for dipeptide repeat protein aggregates and nuclear DNA damage in disease, consistent with experimental studies showing that overexpression of dipeptide repeat proteins are toxic both in cells and in animal models (13–32). While the transcriptome appeared to be shaped in large part by the loss of *C9orf72* function, it is unclear whether the loss of *C9orf72* function contributes to disease and thus understanding whether these changes are functionally linked to disease pathogenesis requires additional experimental studies and validation. Finally, our analysis highlights the complexities associated with molecular studies of *post-mortem* human tissue wherein issues related to cell identity, experimental design, and admixtures of both gain and loss of function effects can be seen concurrently.

## Supplementary Material

Supplementary tables

## Figures and Tables

**Table 1:** Autopsy cohort characteristics. ALS = amyotrophic lateral sclerosis; FTLD = frontotemporal lobar degeneration with TDP-43 inclusions; MND = motor neuron disease; NL = normal; PMI = *post-mortem* interval; RIN = RNA integrity number

Case	Sex	Age of Death	Age of Onset	PMI	Diagnosis	MND	Dementia	RIN
*C9orf72*	F	71	62	3.5	FTLD	N	Y	6.4
*C9orf72*	M	57	55	8.5	FTLD-ALS	Y	Y	7.7
*C9orf72*	F	61	57	12	FTLD	N	Y	6.9
*C9orf72*	F	73	N/A	N/A	FTLD	N	Y	8.2
*C9orf72*	M	77	71	5	FTLD	N	Y	6.2
*C9orf72*	M	57	55	6	FTLD-ALS	Y	Y	7.3
*C9orf72*	M	75	71	10	FTLD-ALS	Y	Y	2.5
Control	M	74		7.5	NL	N	N	7.1
Control	F	82		5	NL	N	N	7.3
Control	M	55		11.5	NL	N	N	6.8
Control	F	61		20	NL	N	N	7.8
Control	F	59		13	NL	N	N	8.2
Control	M	59		17	NL	N	N	6.9

**Table 2:** Gene ontology (GO) of genes related to top 1% of all genes that contribute to variation in PC2.

GO Category	GO Term	No. of Genes	Adj. p-value
Molecular Function	11-beta-hydroxysteroid dehydrogenase [NAD(P)] activity	2	2.18E-2
Cellular Component	Cytoplasmic vesicle part	13	4.02E-2
Cellular Component	Cytoplasmic vesicle membrane	12	4.02E-2

**Table 3:** Annotation of significantly differentially expressed genes with general terms relevant to C9orf72 mutation. FDR = false discovery rate.

Terms of Interest	Gene	Fold Change (log2)	FDR	Name
SYNAPTIC VESICLE / ENDOCYTOSIS	*BIN1*	0.45	0.01	bridging integrator 1
	*DLG4*	0.48	0.04	discs large MAGUK scaffold protein 4
	*SNPH*	0.62	0.01	syntaphilin
	*CLTB*	0.66	0.03	clathrin light chain B
	*SYP*	0.94	0.01	synaptophysin
	*AHNAK*	0.95	0.04	AHNAK nucleoprotein
VESICLE TRANSPORT/ENDOSOMAL TRAFFICKING	*MVB12A*	0.86	0.05	multivesicular body subunit 12A
	*RIN2*	0.68	0.01	Ras and Rab interactor 2
	*SCAMP2*	0.49	0.05	secretory carrier membrane protein 2
	*LRPPRC*	−0.40	0.00	leucine rich pentratricopeptide repeat containing
	*RAB40B*	−0.47	0.01	RAB40B, member RAS oncogene family
LYSOSOMAL INVOLVEMENT	*LYST*	−0.31	0.05	lysosomal trafficking regulator
	*ASGR1*	0.72	0.03	asialoglycoprotein receptor 1
	*CLN3*	0.76	0.04	CLN3, battenin
ALTERNATIVE SPLICING	*CLK4*	−0.66	0.04	CDC-like kinase 4
	*RBM39*	−0.40	0.04	RNA binding motif protein 39
	*ZRSR2*	0.46	0.03	zinc finger CCCH-type, RNA binding motif and serine/arginine rich 2
	*SNRPA*	0.82	0.03	small nuclear ribonucleoprotein polypeptide A
PROTEIN AGGREGATE	*DNAJB11*	−0.87	0.04	DNAJ (Hsp40) homolog, subfamily B, member 11
	*DNAJB2*	0.74	0.03	DNAJ (Hsp40) member B2
	*MGRN1*	0.74	0.00	mahogunin ring finger 1
DNA REPAIR	*HMGN1*	−0.66	0.04	high mobility group nucleosome binding domain 1
	*FANCB*	−0.64	0.04	Fanconi anemia, complementation group B
	*KIN*	−0.35	0.04	Kin17 DNA and RNA binding protein
	*KDM4B*	0.42	0.05	lysine demethylase 4B
	*FAAP20*	0.68	0.03	Fanconi anemia core complex associated protein 20
	*FAM175A*	0.73	0.04	family with sequence similarity 175 member A
